# Estimation of forest aboveground biomass and uncertainties by integration of field measurements, airborne LiDAR, and SAR and optical satellite data in Mexico

**DOI:** 10.1186/s13021-018-0093-5

**Published:** 2018-02-21

**Authors:** Mikhail Urbazaev, Christian Thiel, Felix Cremer, Ralph Dubayah, Mirco Migliavacca, Markus Reichstein, Christiane Schmullius

**Affiliations:** 1 Department of Earth Observation, Institute of Geography, Friedrich-Schiller-University Jena, 07743 Jena, Germany; 2 International Max Planck Research School (IMPRS), Max Planck Institute for Biogeochemistry, 07745 Jena, Germany; 3 Department of Geographical Sciences, University of Maryland, MD 20742 College Park, USA; 4 Department of Biogeochemical Integration, Max Planck Institute for Biogeochemistry, 07745 Jena, Germany

## Abstract

**Background:**

Information on the spatial distribution of aboveground biomass (AGB) over large areas is needed for understanding and managing processes involved in the carbon cycle and supporting international policies for climate change mitigation and adaption. Furthermore, these products provide important baseline data for the development of sustainable management strategies to local stakeholders. The use of remote sensing data can provide spatially explicit information of AGB from local to global scales. In this study, we mapped national Mexican forest AGB using satellite remote sensing data and a machine learning approach. We modelled AGB using two scenarios: (1) extensive national forest inventory (NFI), and (2) airborne Light Detection and Ranging (LiDAR) as reference data. Finally, we propagated uncertainties from field measurements to LiDAR-derived AGB and to the national wall-to-wall forest AGB map.

**Results:**

The estimated AGB maps (NFI- and LiDAR-calibrated) showed similar goodness-of-fit statistics (R^2^, Root Mean Square Error (RMSE)) at three different scales compared to the independent validation data set. We observed different spatial patterns of AGB in tropical dense forests, where no or limited number of NFI data were available, with higher AGB values in the LiDAR-calibrated map. We estimated much higher uncertainties in the AGB maps based on two-stage up-scaling method (i.e., from field measurements to LiDAR and from LiDAR-based estimates to satellite imagery) compared to the traditional field to satellite up-scaling. By removing LiDAR-based AGB pixels with high uncertainties, it was possible to estimate national forest AGB with similar uncertainties as calibrated with NFI data only.

**Conclusions:**

Since LiDAR data can be acquired much faster and for much larger areas compared to field inventory data, LiDAR is attractive for repetitive large scale AGB mapping. In this study, we showed that two-stage up-scaling methods for AGB estimation over large areas need to be analyzed and validated with great care. The uncertainties in the LiDAR-estimated AGB propagate further in the wall-to-wall map and can be up to 150%. Thus, when a two-stage up-scaling method is applied, it is crucial to characterize the uncertainties at all stages in order to generate robust results. Considering the findings mentioned above LiDAR can be used as an extension to NFI for example for areas that are difficult or not possible to access.

## Background

Tropical intact and regrowth forests have the highest carbon (C) uptake of the world’s forests. They account for around 70% of global gross forest sink [1]. At the same time tropical forests are nearly carbon-neutral taking into account C-emissions from tropical deforestation with the highest uncertainties in C-stocks and -fluxes compared to other biomes [1]. The status of tropical forests and their temporal dynamics can be assessed by measuring different structural tree parameters (e.g., vegetation height, canopy cover, stem volume and AGB). AGB, defined as the total amount of aboveground living organic matter in vegetation and expressed as oven-dry tons per unit area [2], is one of the crucial parameter to assess terrestrial aboveground C-stocks and -fluxes. Since vegetation biomass affects a range of ecosystem processes such as carbon and water cycling, energy fluxes, and thus affects local and regional climate, accurate AGB information is required for developing sustainable forest management strategies.

Traditionally, vegetation structural parameters are assessed using forest inventory data. These measurements are demanding in terms of costs and resources, and thus are limited in space and time. With rapid advances in information technology vegetation parameters can be estimated using remote sensing methods. In particular, in tropical forests remote sensing data provide spatially consistent information for areas that are difficult to access. Moreover, in contrast to point measurements spatial continuous AGB maps can improve estimates of carbon flux [3].

In the past 20 years a number of studies aiming at AGB estimation using remote sensing data have been published. These studies reach from local [e.g., 4] over national [5–7] to continental [8] and intercontinental scales [9–12]. In general, remote sensing data from optical, Synthetic Aperture Radar (SAR), and LiDAR sensors or a combination of these sensors are used to estimate AGB. Optical remote sensing data (e.g., Landsat, Sentinel-2, MODIS) are sensitive to vegetation density [5], which relates to AGB but saturates at high biomass [e.g., 13, 14]. Disadvantages in using optical data for AGB estimation are frequent cloud cover over the tropics, and strong dependence on environmental, seasonal and acquisition conditions (e.g., solar zenith angle) [15]. Alternatively, SAR sensors can be used for the estimation of woody vegetation parameters [e.g., 16, 17–24]. For instance, Hame et al. [22] showed that with L-band SAR data estimation of biomass in tropical forests was nearly as good as with optical imagery. Microwave signals (with a spectral range between 1 cm and 1 m) have the capability to penetrate into vegetation, and thus to probe the three-dimensional vegetation structure. Additionally, microwaves are particularly useful for weather independent applications, as long wavelengths penetrate clouds. Limitations of radar data for AGB estimation are saturation at middle-high biomass levels (depending on wavelength) as well as strong dependence on environmental conditions (e.g., rainfall, freezing, different moisture conditions). A way to delineate precise 3D information about the objects on the earth’s surface (trees, buildings) and the topography is the usage of LiDAR. Laser pulses sent from a LiDAR sensor are capable to penetrate forest canopy, and to provide information on the vertical structure (e.g., height, canopy volume). LiDAR data can be used to delineate very accurate estimates of AGB without signal saturation. Accordingly, LiDAR is a key information source for assessing carbon stocks including tropical forests [25]. Zolkos et al. [26] compared more than 70 studies for AGB estimation and concluded that airborne LiDAR methods provide a higher accuracy compared to SAR or optical data. However, airborne LiDAR data is limited to a small spatial coverage.

The signals from optical, SAR, or LiDAR sensors are commonly compared to the field-estimated AGB using semi-empirical regression models or machine learning algorithms to extrapolate over the entire remote sensing imagery. As mentioned above the plot estimates of AGB are limited in time and space, and might thus not represent the full spectrum of vegetation types or AGB [27]. Alternatively, very high resolution (VHR) (< 2 m) remote sensing data from airborne LiDAR or optical sensors can be used as reference data for up-scaling to larger area. Currently, many large scale mapping efforts both for AGB estimation and forest cover delineation have been applied a two-stage up-scaling method (i.e., from field measurements to LiDAR strips or VHR optical imagery and from LiDAR-, VHR-based estimates to satellite imagery) [9, 10, 28–30]. One important step in the two-stage up-scaling method is error propagation analysis. As showed in [31, 32], ignoring the field to LiDAR error can underestimate the uncertainty in the final satellite-based AGB map by a factor of three or more. Therefore, an uncertainty map at pixel level is important for the interpretation of the AGB map.

In this study, we estimated forest AGB in Mexico at national scale, where both extensive NFI (~ 15,000 plots) (Spanish acronym INFyS) and country-wide airborne LiDAR data were available. As spatial predictors to estimate AGB over Mexico we used satellite imagery from the Advanced Land Observing Satellite Phased Array type L-band Synthetic Aperture Radar (ALOS PALSAR), Landsat and the Shuttle Radar Topography Mission (SRTM), since a fusion of optical and SAR imagery provides more accurate estimates of AGB compared to single sensor type data [6, 7, 33, 34]. We estimated AGB at national scale using two modelling scenarios: (1) using INFyS data collected over the country with systematic sampling as calibration data for satellite imagery, (2) using airborne LiDAR-based AGB as calibration data for satellite imagery. Both national AGB products were validated with INFyS data that were not used for model calibration. Furthermore, we conducted an error propagation analysis for both scenarios and estimated uncertainties at pixel level using Monte Carlo simulations. This kind of comprehensive comparison between NFI and LiDAR data as reference for a large scale AGB mapping with satellite imagery including an error propagation analysis have not been conducted before. This gap needs to be addressed, especially in the context of the upcoming missions designed for global vegetation monitoring (e.g., NISAR, GEDI, BIOMASS, Tandem-L).

## Methods

### Study area and field data

**Fig. 1:**
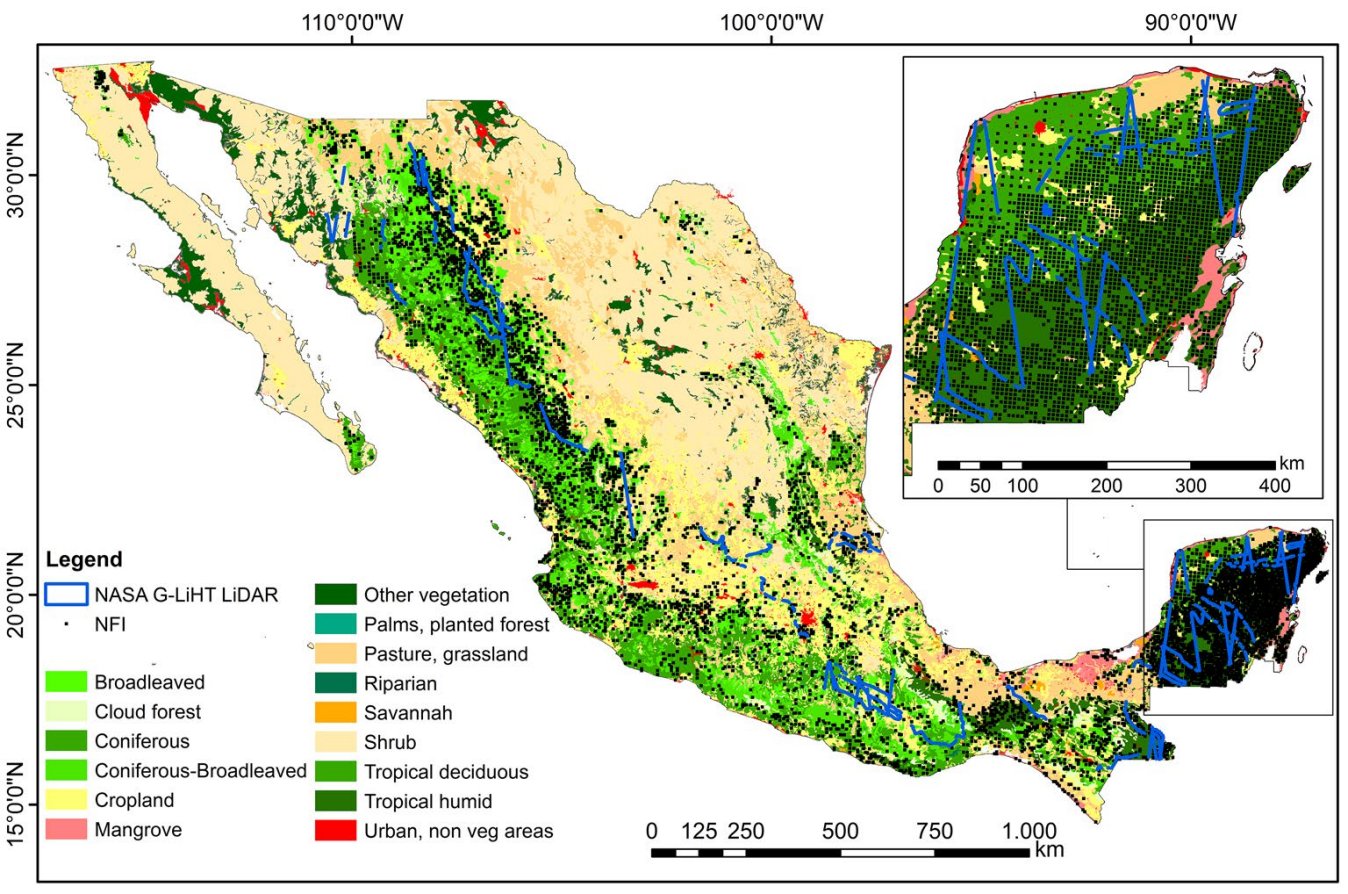
Land use and vegetation map of Mexico from the Mexican National Institute for Statistics and Geography (INEGI) Series IV [61]

Approximately one-third of Mexico is covered by forests resulting in 65 million ha [35] with a variety of forest types (deciduous and coniferous forests, mangroves, cloud forests, and tropical dry and rain forests) (Fig. 1). These forests are located at different topographies (from coastal plain in the Yucatan peninsula to mountainous regions in central part of the country).

**Fig. 2:**
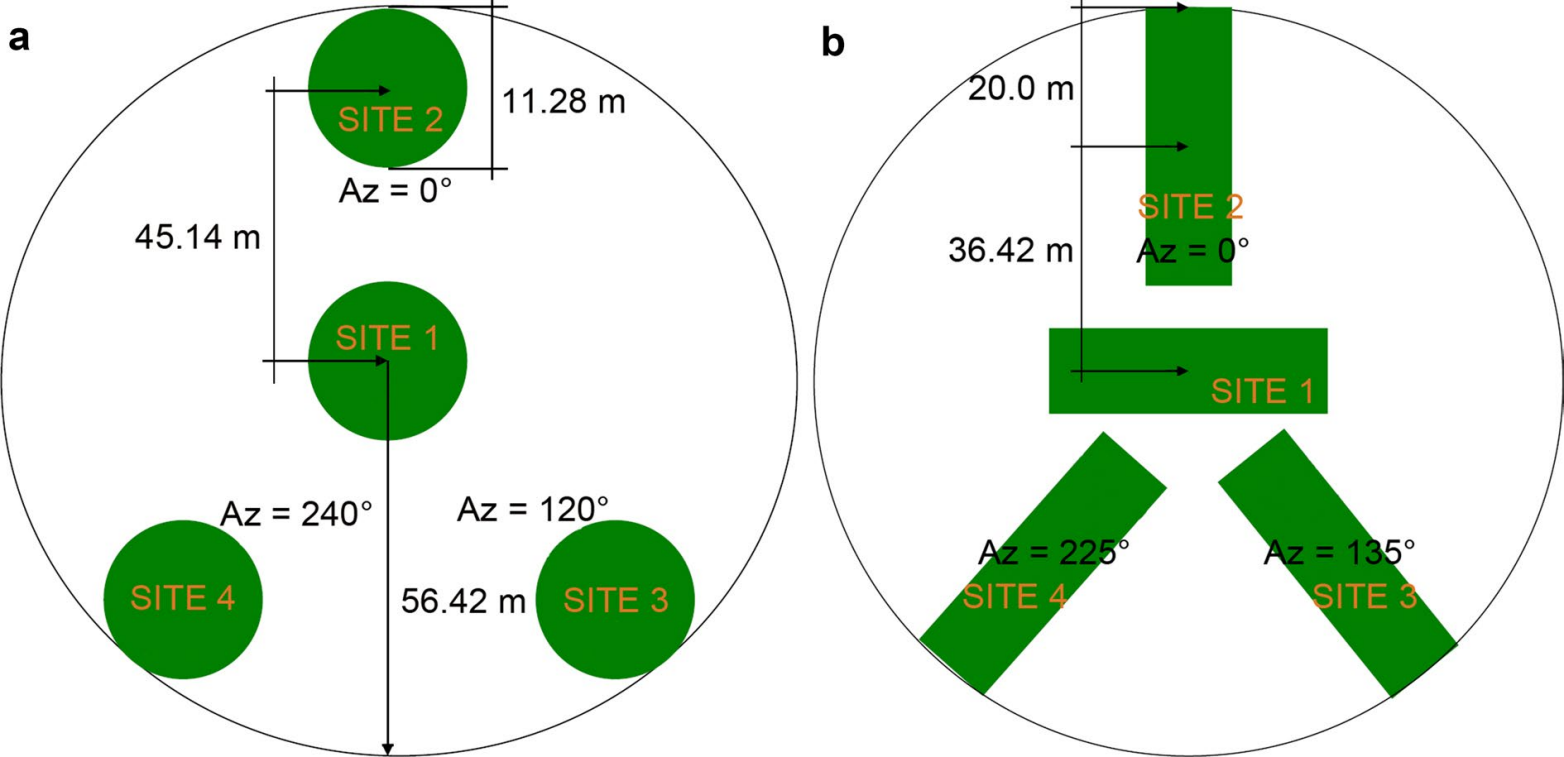
INFyS sampling plot design for **a** temperate and **b** tropical forests

The National Forestry Commission of Mexico (CONAFOR) has established a systematic nationwide network of forest inventory plots (Fig. 1). In this study, NFI data collected between 2004 and 2011 were used. One sampling plot represents a single circular plot with a radius of 56.42 m covering an area of 1 ha and comprising four sub-plots with an area of 400 m^2^ each (0.04 ha). For temperate and tropical forests different sampling designs were used (Fig. 2). Each circular plot was sampled using rectangular grid with a distance between single plots varying from 5 km (tropical/temperate forests) to 20 km (arid regions) resulting in 28,869 plots, while most of the plots were sampled twice during the mentioned 7 year period. Within each sub-plot different structural tree parameters (e.g., diameter at breast height, mean tree height etc.) were measured. AGB was calculated for each sub-plot (total sampled area of 0.16 ha) using 339 species- and genus-specific allometric models and wood densities [36] and then extrapolated to 1 ha. From all available INFyS data, plots with less than four sub-plots measurements were discarded (1786 plots). Further, for the plots comprising two temporal measurements (either 2004–2007 or 2008–2011) the temporal average was calculated. This step was conducted in order to reduce imprecision due to geolocation errors or inaccurate measurements resulting in 15,982 plots. Finally, inventory plots located on steep slopes (> 15°) were also excluded from the analysis (8441 plots), as they can be located in SAR layover and shadow areas and often show high geolocation errors. In total 7541 forest inventory plots were used for AGB mapping and product validation. From 7541 plots, 332 plots were used for AGB estimation along the LiDAR strips. The remaining 7209 field plots were divided into calibration (67%) and validation (33%) data sets based on biomass intervals. For this, the NFI data set were split into ten biomass classes with an interval of 30 t/ha, 67% from each class were selected randomly for calibration and the remaining plots were used for validation. An overview of the whole procedure can be found in Fig. 4.

### Remote sensing data

#### Airborne LiDAR data

Small footprint discrete-return airborne LiDAR data were collected by NASA’s G-LiHT imager [37] in April–May 2013 over the entire country resulting in 1123 strips (Fig. 1). The average pulse density was approximately 6 returns/m^2^. The data were acquired during leaf-off conditions. From the topography-normalized point clouds 88 plot-aggregated LiDAR metrics as described in [38–40] were calculated at 1 ha scale. These LiDAR metrics correspond to the vertical structure of a target and were used as predictor variables to estimate AGB along the LiDAR strips (“Estimation of errors in the field-estimated AGB” section).

#### Satellite imagery

In our study, we used ALOS PALSAR L-band SAR and Landsat optical data. The L-band SAR data were collected and processed by Japan Aerospace Exploration Agency (JAXA) in dual-polarization mode (i.e., HH/HV polarizations). The JAXA pre-processed ALOS PALSAR backscatter (gamma nought) mosaics were slope-corrected and orthorectified using a digital elevation model (DEM) [41, 42]. The mosaics feature a pixel spacing of 25 m × 25 m and are provided for free [43]. In the next step, ALOS PALSAR backscatter images were speckle filtered using the multi-temporal filter after Quegan et al. [44, 45] with a window size of 7 × 7 pixels. In order to evaluate the amplitude of speckle, the equivalent number of looks (ENL) was calculated over homogeneous areas for original and filtered images using an empirical approach after [46] (i.e., ENL = mean^2^/variance). The ENL was increased by factor 2 both for HH and HV polarizations indicating a reduction of speckle.

Optical data was used in form of spectral reflectance (SR) mosaic based on Landsat 5 and 7 ETM + data for the year 2012. This Landsat SR mosaic was published by Hansen et al. [47] and is freely accessible [48]. From the Landsat SR the Normalized Differenced Vegetation Index (NDVI) was calculated and used as a predictor layer. A further predictor layer was the Landsat tree cover product by Hansen et al. [47] for the year 2010. First independent product validations suggest that this tree cover product features high accuracy. For instance, a validation study conducted over South America based on VHR commercial optical imagery showed a strong agreement with an R^2^ of 0.82 [49]. Finally, altitude and slope information obtained from the Shuttle Radar Topography Mission (SRTM) DEM data version 4.1 [50] were utilized in AGB modelling. All spatial data sets were aggregated to 100 m pixel size using block averaging and nearest neighbour resampling. In total, 16 predictor layers were used for AGB estimation (“AGB modelling and uncertainty analysis” section) (Table 1).

**Table 1:** Remote sensing products used for AGB estimation at national scale

Remote sensing product	Spatial resolution	Acquisition date	Layers
ALOS PALSAR	25 m	2007–2010	SAR backscatter: HH and HV polarization for 2007–2010
Landsat	30 m	2010–2012	Normalized Top-of-atmosphere (TOA) Reflectance: band 3 (red), Band 4 (NIR), Band 5 (SWIR), Band 7 (SWIR); NSVI; tree cover
SRTM DEM	30 m	2000	Altitude; slope

### AGB modelling and uncertainty analysis

**Fig. 3:**
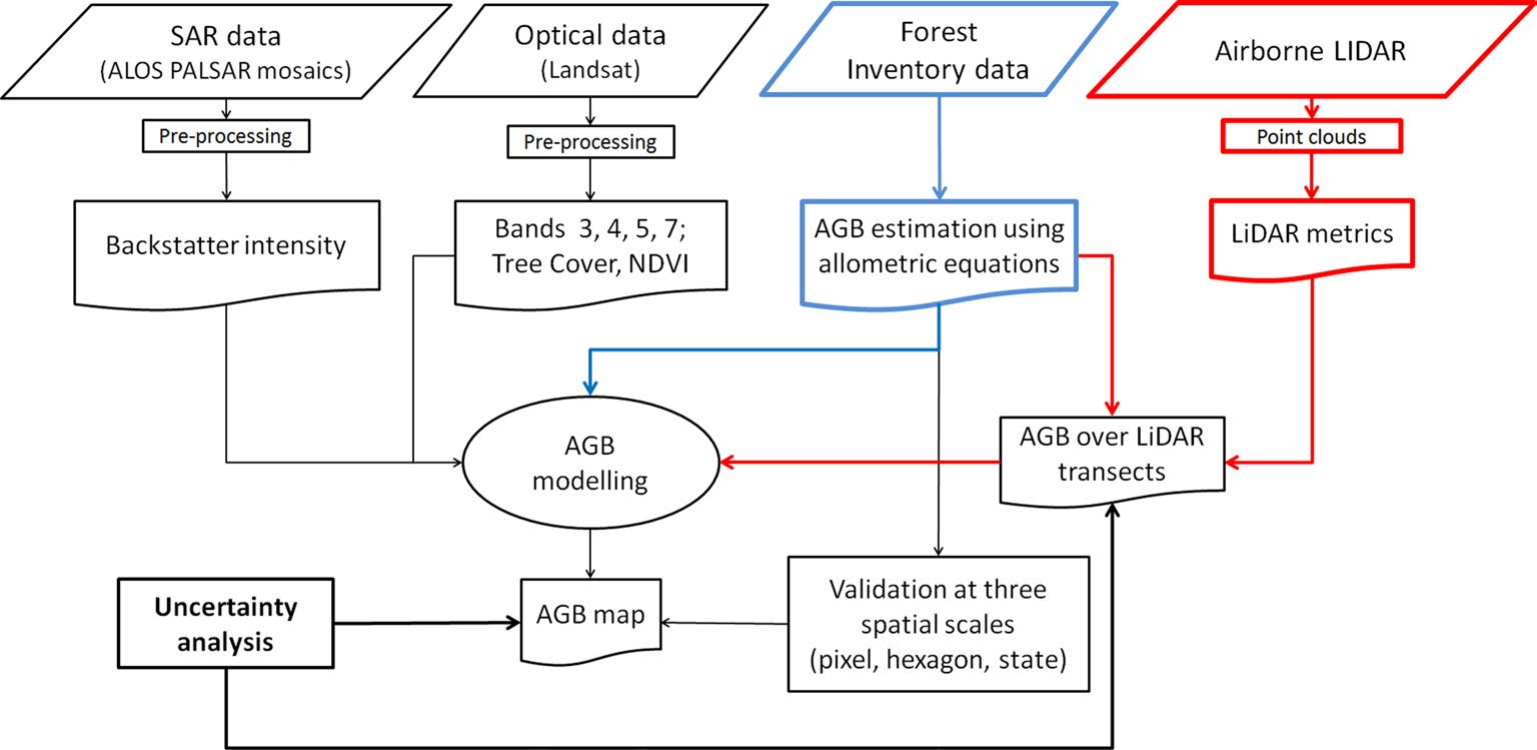
Flow chart of the data processing and analysis steps. Blue: first modelling scenario based on NFI data; red: second modelling scenario based on two-stage up-scaling method

As mentioned above, two modelling scenarios were applied. As NFI data were collected over the whole country, we developed a model that was calibrated with NFI data only (scenario 1, “Estimation of AGB and uncertainties at national scale with NFI-AGB as calibration data” section). In the other scenario, we applied a two-stage up-scaling method (i.e., from field measurements to LiDAR strips and from LiDAR-based estimates to satellite imagery) (Fig. 3) (scenario 2, “Estimation of AGB and uncertainties along the LiDAR strips”, “Estimation of AGB and uncertainties at national scale with LiDAR-AGB as calibration data” section). Since NFI data were collected over forested areas only, we applied a forest mask to the wall-to-wall AGB maps. For this task, the Landsat tree cover product for 2010 [47] was used (forest = tree cover > 10% according to FAO definition of forest [51]).

We estimated uncertainties at pixel level for both scenarios using Monte Carlo simulations. For this, we introduced an error term in field-estimated AGB (“Estimation of errors in the field-estimated AGB” section) and propagated it to satellite-estimated AGB (“Estimation of AGB and uncertainties at national scale with NFI-AGB as calibration data” section). In the two-stage up-scaling method, first we propagated errors of field-estimated AGB to LiDAR-estimated AGB (“Estimation of AGB and uncertainties along the LiDAR strips” section) and the latter to satellite-estimated AGB (“Estimation of AGB and uncertainties at national scale with LiDAR-AGB as calibration data” section).

#### Estimation of errors in the field-estimated AGB

The total error of the field-estimated AGB (*ɛ*_*field*_) was composed of three components which were assumed to be independent and random and were calculated as follows:

1 $$\varepsilon_{field} = \left( {\varepsilon_{measurement}^{2} + \varepsilon_{allometry}^{2} + \varepsilon_{samplng}^{2} } \right)^{1/2}$$

where *ɛ*_*measurement*_, *ɛ*_*allometry*_, and *ɛ*_*sampling*_ are the measurement error of tree parameters (e.g., diameter at breast height (dbh) and tree height), allometric model error, and sampling error, respectively. Chave et al. [52] estimated the measurement error of individual trees in central Panama to be 16%. As it averages out at stand level [52], it was assumed to be 10% in this study [53]. For species-specific allometric models, we assumed an error of 11% [52]. To estimate the sampling error, we approximated the errors using the study from [54]. In the study in central Panama, the authors concluded that in order to estimate AGB for a 50 ha plot with ± 10% uncertainty at least 160 of 0.04 ha plots are needed [54]. This requires a sampling intensity of 12.8%. By assuming similar variability in 1 ha pixel, and thus similar sampling intensity, the number of 0.04 ha plots required to estimate AGB with ± 10% uncertainty will be 3.2. Therefore, the sampling error in our study was 8.9% $\left( {10\, \times \,\sqrt {3.2/4} } \right)$. By summing up each single error term, we suggest that our field-estimated AGB have an error of around 17%.

Under the assumption that our field-estimated AGB (*Field*_*AGB*_) have an error of 17%, we generated 100 realizations of field-estimated AGB ($\widehat{{Field_{AGB} }}$) using normally distributed random values:

2 $$\widehat{{Field_{{AGB_{i,j} }}^{n} }} = Field_{{AGB_{i,j} }} \times \left( {1 + \varepsilon_{field} \times X_{i,j}^{n} } \right)$$

where the symbol “ˆ” denotes a variable that includes the estimated error, *n* is number of Monte Carlo realizations, *i*, *j* is a single pixel, *X* is a random number from a normal distribution with mean = 0 and standard deviation = 1.

#### Estimation of AGB and uncertainties along the LiDAR strips

To estimate AGB along the LiDAR strips, all NFI data that were located completely within the LiDAR data were selected (332 plots). Since the difference in acquisition time between NFI and LiDAR data is between 2 and 9 years, significant changes (caused, e.g., by fire or deforestation) within the field plots might have occurred [33]. Consequently, plots for which the residuals exceeded a range of two times the residual standard deviation (20 plots) were excluded from the analysis. For these 312 field plots 100 Monte Carlo realizations of field-estimated AGB $\widehat{{(Field_{AGB} )}}$ were generated and used as response variable. As spatial predictors 88 plot-aggregated LiDAR metrics (*LiDAR*_*metrics*_) were used. We estimated 100 different LiDAR-AGB calibrated with $\widehat{{Field_{AGB} }}$ using a machine learning approach *Cubist*. *Cubist* is a hybrid tree-based approach that combines rule-based regression with linear multivariate models. Based on the training data a collection of rules is defined. A rule represents a path through a decision tree, for each rule a multivariate linear regression is used to calculate a predicted value. The final prediction is calculated by combining linear models at each node of the trees; therefore, it is smoothed compared to a single linear model. The approach is described in Quinlan [55, 56]. Cubist is computational efficient and robust non-parametric model and was successfully applied to map vegetation structure metrics (e.g., AGB, tree height) with high retrieval accuracy at large spatial scales [57–60].

The 100 LiDAR-AGB estimations for each pixel $\widehat{{\left( {LiDAR_{{AGB_{i,j} }} } \right)}}$ were calculated:

3 $$\widehat{{LiDAR_{{AGB_{i,j} }}^{n} }} = cubist\left( {\widehat{{FieldAGB_{i,j}^{n} }},LiDAR_{metrics} } \right)$$

From these 100 LiDAR-AGB realizations, 95% confidence interval (CI 95) was calculated:

4 $$CI95 \left( {\widehat{{LiDAR_{{AGB_{i,j} }}^{n} }}} \right) = \frac{{CI_{97.5} - CI_{2.5} }}{2}$$

The uncertainty for each LiDAR-AGB pixel was calculated:

5 $$\varepsilon_{LiDAR} = \frac{{CI 95 \left( {\widehat{{LiDAR_{{AGB_{i,j} }}^{n} }}} \right)}}{{mean\left( {\widehat{{LiDAR_{{AGB_{i,j} }}^{n} }}} \right)}} \times 100$$

#### Estimation of AGB and uncertainties at national scale with NFI-AGB as calibration data

For the first modelling scenario at national scale (i.e., based on NFI data only), we proceed similar as for the estimation of AGB along the LiDAR strips. The estimation of AGB at national scale was performed using a machine learning algorithm *Cubist* [55, 56]. As response variable we used 100 Monte Carlo realizations of NFI-estimated AGB ($\widehat{{Field_{AGB} }}$), while satellite data (*Sat*_*layers*_) (“Satellite imagery” section, Table 1) were used as spatial predictors. 100 AGB maps at national scale based on the first modelling scenario ($\widehat{{Sat\_NFI_{AGB} }}$) were estimated as:

6 $$\widehat{{Sat\_NFI_{{AGB_{i,j} }}^{n} }} = cubist\left( {\widehat{{Field_{{AGB_{i,j} }}^{n} }},Sat_{layers} } \right)$$

Based on the 100 NFI-calibrated national AGB estimates ($\widehat{{Sat\_NFI_{AGB} }}$) the 95% confidence interval ($CI 95 \widehat{{\left( {Sat\_NFI_{{AGB_{i,j} }}^{n} } \right)}}$) was calculated (Eq. 4), and the uncertainty for each pixel was determined:

7 $$\varepsilon_{Sat\_NFI} = \frac{{CI 95 \widehat{{\left( {Sat\_NFI_{{AGB_{i,j} }}^{n} } \right)}}}}{{mean\widehat{{\left( {Sat\_NFI_{{AGB_{i,j} }}^{n} } \right)}}}} \times 100.$$

Finally, we applied the Landsat tree cover product from 2010 [47] to mask areas covered by forests.

#### Estimation of AGB and uncertainties at national scale with LiDAR-AGB as calibration data

In the second modelling scenario at national scale, a two-stage up-scaling method was applied. Similar to the first modelling scenario, we applied the machine learning algorithm *Cubist* [55, 56] and used the same satellite imagery (*Sat*_*layers*_) as spatial predictors (Table 1). As model calibration data we used 100 LiDAR-AGB estimations ($\widehat{{LiDAR_{AGB} }}$) that already include the estimated error of field data and the model prediction error for the LiDAR strips. Accordingly, 100 AGB maps at national scale based on second modelling scenario ($\widehat{{Sat\_LiDAR_{AGB} }}$) were estimated as:

8 $$\widehat{{Sat\_LiDAR_{{AGB_{i,j} }}^{n} }} = cubist\left( {\widehat{{LiDAR_{{AGB_{i,j} }}^{n} }},Sat_{layers} } \right).$$

Again, based on the 100 LiDAR-calibrated national AGB estimates ($\widehat{{Sat\_LiDAR_{AGB} }}$) the 95% confidence interval ($CI 95 \left( {\widehat{{Sat\_LiDAR_{{AGB_{i,j} }}^{n} }}} \right)$) was calculated (Eq. 4) and the uncertainty for each pixel was determined:

9 $$\varepsilon_{Sat\_LiDAR} = \frac{{CI 95 \widehat{{\left( {Sat\_LiDAR_{{AGB_{i,j} }}^{n} } \right)}}}}{{mean\widehat{{\left( {Sat\_LiDAR_{{AGB_{i,j} }}^{n} } \right)}}}} \times 100.$$

**Fig. 4:**
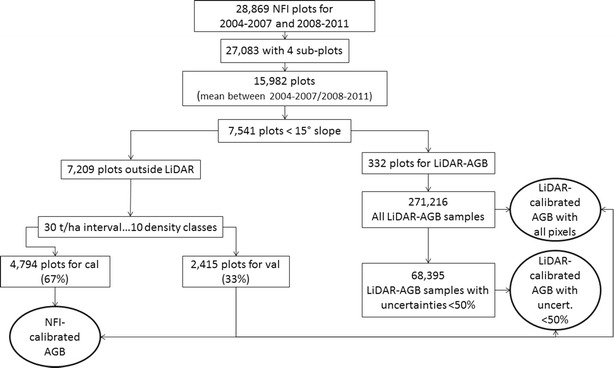
Filtering steps of reference data (both NFI and LiDAR) for calibration of satellite imagery and validation of the AGB maps

Additionally to the modelling scenario based on all LiDAR-AGB estimates, we estimated AGB at national scale using LiDAR-AGB samples with uncertainties below 50% $\left( {Sat\_LiD\widehat{{AR_{{AGB_{uncert50} }} }}} \right)$. This modelling scenario was conducted in order to prevent the propagation of high uncertainties of the LiDAR-AGB to the final AGB map. The threshold of 50% is a trade-off between retaining LiDAR samples for training and keeping the uncertainties of the wall-to-wall map at a low level (i.e., a lower threshold will lead to a lower number of training data; a higher threshold will lead to higher uncertainties in the wall-to-wall map). The number of remaining LiDAR-AGB samples can be found in Fig. 4. In the next step, we estimated the uncertainties for AGB map calibrated with LiDAR-AGB pixels with uncertainties below 50% (*ɛ*_*Sat*_*LiDARuncert*50_) (Eq. 9).

Eventually, all non-forest areas were discarded again using the Landsat tree cover product from 2010 [47].

### Validation of mean forest AGB maps at different scales

Both national AGB maps (based on NFI and LiDAR training data) were validated at pixel level. For each modelling scenario (i.e., NFI- and LiDAR-calibrated AGB models), we calculated a mean AGB value from 100 Monte Carlo realizations. Goodness-of-fit statistics (R^2^, RMSE, bias) were calculated between NFI- and LiDAR-calibrated mean AGB and the validation data set (“Study area and field data” section) (Fig. 4).

The validation was also performed at hexagon and state levels. Accordingly, we built a mesh of hexagons over the country with an area of 650 km^2^/hexagon. For each hexagon the modelled AGB (i.e., average of 100 Monte Carlo realizations) and the AGB based on forest inventory were extracted. The percentage of the forest cover per hexagon was considered using as a weighting factor. The forest areas were obtained from the Mexican National Institute for Statistics and Geography (INEGI) Land use map [61], since this map was used to establish the field plots. The national INEGI Land use map was generated using visually interpretation of SPOT optical imagery and field verification at a scale of 1:250,000.

For the validation at state level a similar procedure was applied. For each federal state, AGB values from the modelled maps and NFI plots were extracted and weighted by the forest area delineated from the INEGI Land use map [61]. Finally, linear regressions and statistics (R^2^, RMSE, bias) comparing modelled and field-estimated AGB were calculated at hexagon and state levels.

## Results

### Estimation of AGB and uncertainties along the LiDAR strips

**Fig. 5:**
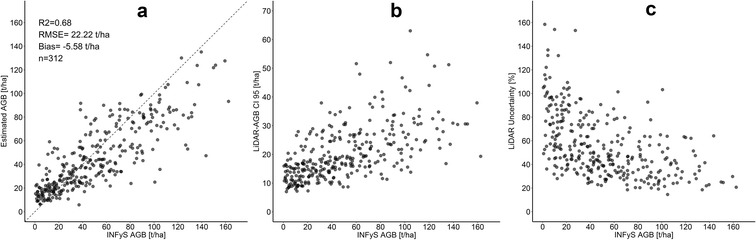
**a** Linear regression between reference (INFyS) and predicted (LiDAR) AGB. The dotted line is the 1:1 line. **b** CI 95 of LiDAR-estimated AGB increases with increasing reference AGB. **c** The highest uncertainties of LiDAR-estimated AGB at low AGB range decreases with higher reference AGB

In order to apply a two-stage up-scaling method (“Estimation of AGB and uncertainties at national scale with LiDAR-AGB as calibration data” section), we first estimated AGB along the LiDAR strips with the *Cubist* machine learning algorithm. We propagated the estimated field error (17%) to the AGB modelling running 100 Monte Carlo simulations (Eq. 3). From the 100 AGB estimations, we calculated the mean AGB, CI 95, and the uncertainty for each single LiDAR pixel and plotted the simulation results against field-estimated AGB (Fig. 5a–c).

**Fig. 6:**
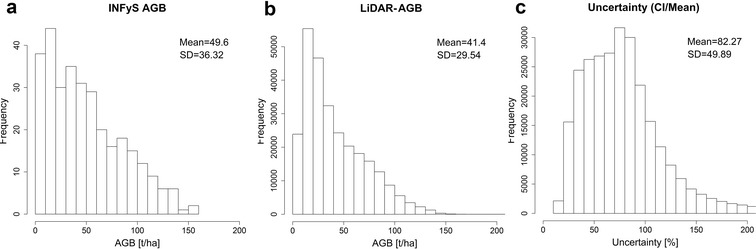
**a** Histogram of field-estimated AGB used for calibration of LiDAR metrics; **b** histogram of LiDAR-estimated AGB; **c** histogram of uncertainties in LiDAR-estimated AGB

Although the correlation between the mean estimated AGB (from Monte Carlo) and field-estimated AGB was strong (Fig. 5a), the uncertainties in the LiDAR-AGB were very high and went up to 200% (Figs. 5c, 6c). The absolute AGB uncertainties (in our case CI 95) increased with increasing AGB (Fig. 5b). However, the highest relative uncertainties were found in areas with low biomass (Fig. 5c), as in these areas small absolute deviations easily result in large relative uncertainties (Eq. 5).

Discrepancies between modelled and reference data are caused by different factors. First, the high variations in 100 LiDAR-AGB estimates (expressed in CI 95 and uncertainties) can be caused by a low amount of training data (i.e., 312 field plots were used to extrapolate AGB for more than 270,000 LiDAR samples), so that each model run produced diverse results. Second, the time lag between the acquisitions of LiDAR and NFI data was between 2 and 9 years, which introduces the potential for changes between both data acquisitons. Third, the sampled area of NFI data of 0.16 ha was extrapolated to 1 ha, i.e., for some plots an area of 0.16 ha may be not representative for the 1 ha plot. Fourth, small inventory plots (4 subplots with 0.04 ha size) are more affected by geolocation errors, since they may not reflect the spatial variability in the surrounding area. As reported in [26], the errors in LiDAR-estimated AGB decrease exponentially with a decreasing plot size, due to spatial averaging of errors [62]. Finally, a universal AGB model developed for different forest types can produce additional errors in the prediction results.

Field-estimated AGB (used for calibration of LiDAR metrics) and the modelled mean LiDAR-AGB showed similar distribution with a mean AGB around 40–50 t/ha, a standard deviation (SD) of 30–40 t/ha, and a maximum AGB up to 150–160 t/ha (Fig. 6a, b). In the mean LiDAR-AGB, however, there were fewer pixels featuring a low AGB (e.g., less than 10 t/ha) compared to the field-estimated AGB. This is caused by the ensemble model of decision trees [34, 63, 64], where single predictions of each tree are averaged. These models in general tend to shift the lowest and highest values towards the mean.

As mentioned above (“Estimation of AGB and uncertainties at national scale with LiDAR-AGB as calibration data” section), we used LiDAR-AGB samples as calibration data for satellite imagery. For this, we used all LiDAR-AGB samples (271,216 1 ha LiDAR samples) as well as LiDAR-AGB pixels with uncertainties below 50% (68,395 1 ha LiDAR samples).

### Estimation of AGB and uncertainties at national scale with NFI-AGB as calibration data

Based on the satellite imagery, the 4794 NFI-estimated AGB samples, and the *Cubist* machine learning algorithm around 65 Mio. ha of forest land was mapped at 1 ha scale. We propagated the estimated field error (17%) in the AGB modelling with 100 Monte Carlo simulations (Eq. 6).

**Fig. 7:**
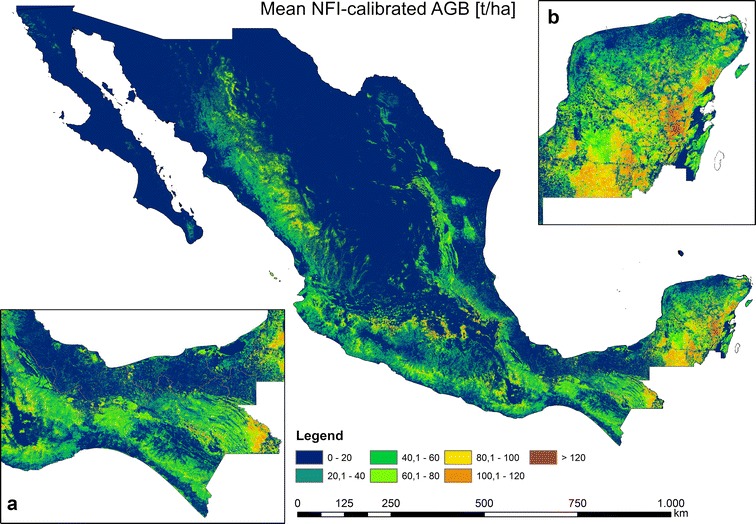
National forest AGB map based on NFI-estimated AGB, satellite imagery, Cubist machine learning algorithm, and Monte Carlo analyses

**Fig. 8:**
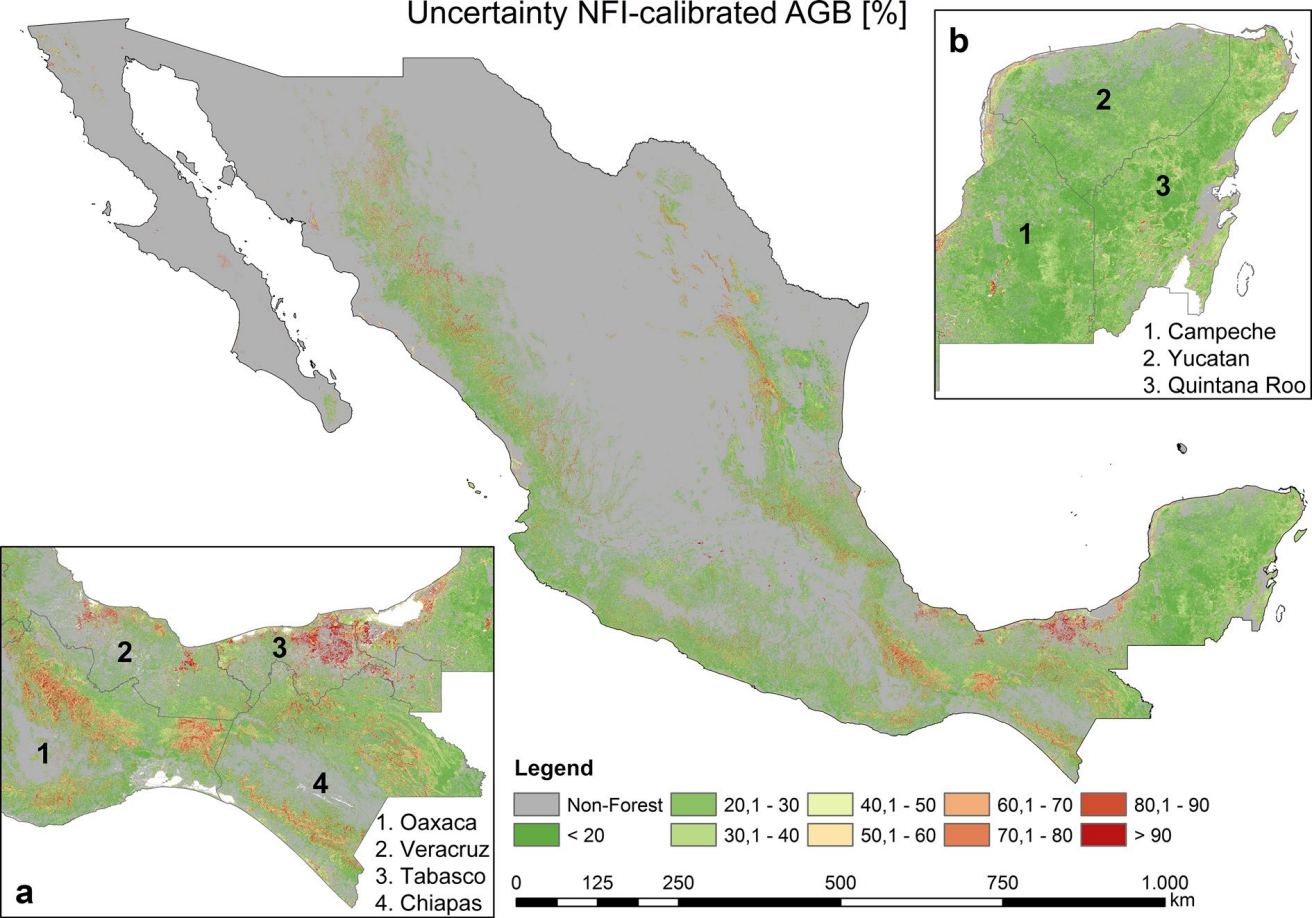
Estimated uncertainties (incl. error in field data and model prediction error) based on NFI-estimated AGB, satellite imagery, Cubist machine learning algorithm, and Monte Carlo analyses

**Fig. 9:**
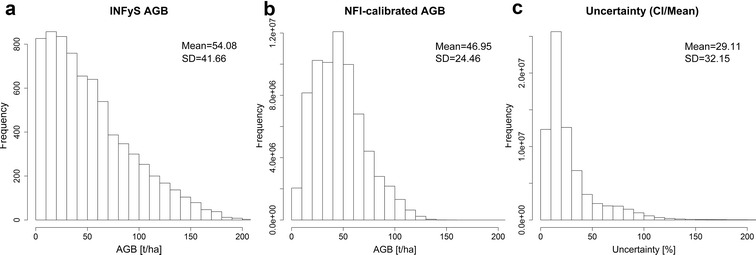
**a** Histogram of field-estimated AGB used for calibration of satellite imagery; **b** histogram of estimated national forest AGB calibrated with field-AGB; **c** histogram of uncertainties in AGB map calibrated with field-AGB

From the 100 AGB estimations, we calculated mean AGB (Fig. 7), CI 95, and uncertainties (Fig. 8) for each single 1 ha pixel. We attributed the last class as AGB > 120 t/ha, since a signal saturation of SAR and optical data for a high AGB range occurred [5, 53, 65, 66], and a relatively small country area possess AGB values higher than 120 t/ha (Fig. 9a). In accordance to [6, 7], the highest forest AGB were located in the tropical forests of the Yucatan Peninsula (Fig. 7b) and Chiapas (Fig. 7a) as well as in the mountain forests of Trans-Mexican Volcanic Belt (close to Mexico City). Since we applied a forest mask with 10% tree cover, the AGB in north-central parts of Mexico can be underestimated. The total forest aboveground carbon (AGC) was found to be 1.602 PgC (conversion factor of 0.48). This value is close to the Mexican forest carbon stock according to FAO’s Forest Resource Assessment 2010 (1.688 PgC) [67]. The validation of the map at different scales is presented in section “Validation of forest AGB maps at different scales”.

Most forest areas in Mexico possessed AGB uncertainties lower than 20–30% with a mean of 29.11% (Figs. 8, 9c). The areas with the highest AGB uncertainties were found in the states of Oaxaca, Chiapas, and Tabasco (Fig. 8a). For instance, in the state of Tabasco the highest uncertainties (higher than 90%) were estimated for mangrove forests in *Pantanos de Centla* (Fig. 8a). In the states of Oaxaca and Chiapas the highest uncertainties (up to 90%) occurred in the dense cloud forests of *Sierra Madre del Sur* and *Chimalapas* tropical forests, respectively. In contrast, the dense tropical forests of the Yucatan peninsula featuring high forest AGB (Fig. 7b) showed relatively low uncertainties (Fig. 8b) ranging between 20 and 40%. One reason for the low uncertainties is related to the dense NFI network covering the entire peninsula.

Similar to the AGB estimation along the LiDAR strips, the AGB distribution in the national NFI-calibrated map was different at low and high AGB ranges compared to the field-estimated AGB (Fig. 9a, b). This is again partly caused by the characteristics of the ensemble model of decision trees [34, 63, 64] (see above). Also, SAR and optical imagery reached saturation level at high AGB (> 100 t/ha) [5, 53, 65, 66]. The uncertainties in the national NFI-calibrated AGB map were smaller compared to the LiDAR-AGB (Figs. 6c, 9c). These lower variations can be caused by the fact that for the mapping at national scale a much larger reference data set were available compared to the AGB mapping along the LiDAR strips (4794 plots vs. 312 plots). Accordingly, the regression models become more robust.

### Estimation of AGB and uncertainties at national scale with LiDAR-AGB as calibration data

**Fig. 10:**
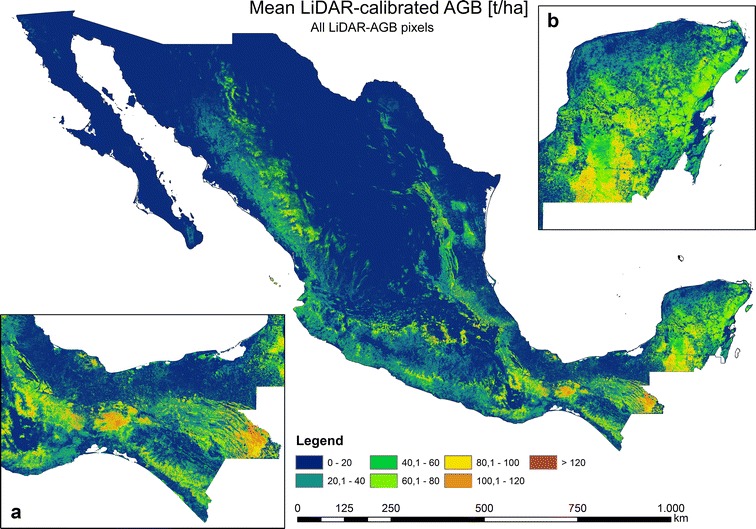
National forest AGB map based on LiDAR-estimated AGB, satellite imagery, Cubist machine learning algorithm, and Monte Carlo analyses

Using the same satellite imagery as for the first modelling scenario (“Estimation of AGB and uncertainties at national scale with NFI-AGB as calibration data”, “Estimation of AGB and uncertainties at national scale with LiDAR-AGB as calibration data” sections) and 271,216 LiDAR-estimated AGB values as calibration data, we applied the *Cubist* machine learning algorithm to map forest AGB in Mexico at 1 ha scale (Fig. 10). Similar to the NFI-calibrated AGB map, the highest AGB in the LiDAR-calibrated map occurred in the Yucatan Peninsula, Chiapas and Trans-Mexican Volcanic Belt. However, in contrast to the NFI-calibrated map, one of the areas featuring the highest AGB was located in the *Chimalapas* and *Lacandon* tropical forests (Figs. 8a, 10a). Furthermore, the spatial AGB pattern in the Yucatan peninsula shows clear differences between both maps (Figs. 8b, 10b, 18). Since we applied a forest mask with 10% tree cover, the AGB in north-central parts of Mexico can be underestimated. The total forest aboveground carbon (AGC) was estimated to be 1374 PgC (conversion factor of 0.48), and thus lower compared to the NFI-calibrated AGB map (1602 PgC) as well as compared to the Mexican forest carbon stock according to FAO’s Forest Resource Assessment 2010 (1.688 PgC) [67]. The validation of the map at different scales is presented in section “Validation of forest AGB maps at different scales”.

**Fig. 11:**
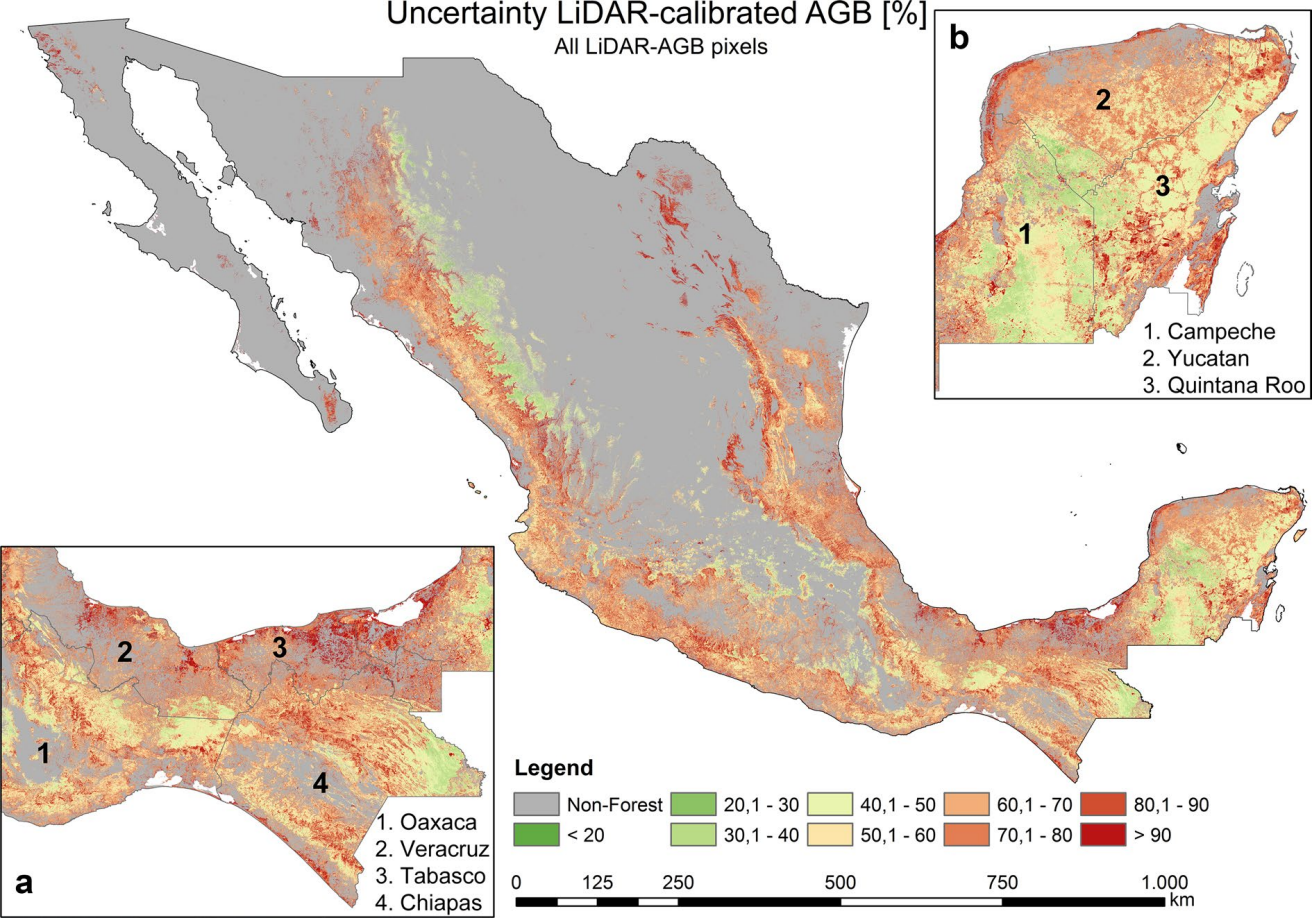
Estimated uncertainties (incl. error in field data, model prediction errors: NFI to LiDAR and LiDAR to satellite imagery) based on all LiDAR-estimated AGB, satellite imagery, Cubist machine learning algorithm, and Monte Carlo analyses

**Fig. 12:**
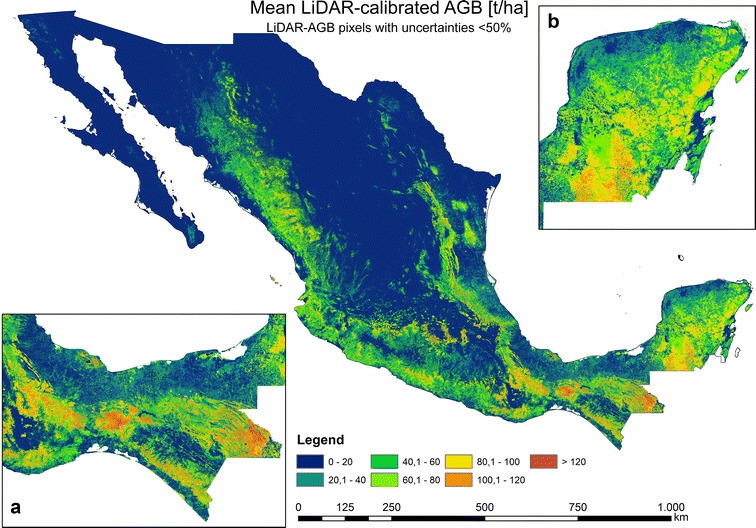
National forest AGB map based on LiDAR-estimated AGB (with uncertainties < 50%), satellite imagery, Cubist machine learning algorithm, and Monte Carlo analyses

When using all LiDAR-AGB values (i.e., with uncertainties up to 200%), the uncertainties in LiDAR-AGB propagated to the final AGB map. Accordingly, the national forest AGB map based on all LiDAR-AGB featured high uncertainties (Fig. 11). Most forest areas in Mexico showed uncertainties between 60 and 90% with a mean of 65.86% (Fig. 12c). High uncertainties (> 60%) occurred in areas with low forest AGB (< 60–80 t/ha), while in areas with high forest AGB (> 80 t/ha) the AGB uncertainties were lower (20–40%) (Figs. 10, 11).

In the next modelling scenario we used only LiDAR-AGB pixels with uncertainties below 50% (henceforth LiDAR-AGB_50%). The majority of the pixel with uncertainties > 50% were located in areas with low forest AGB (“Estimation of AGB and uncertainties along the LiDAR strips” section). For this reason, the forest AGB map calibrated with LiDAR-AGB_50% possessed higher AGB values than the map calibrated with all LiDAR-AGB pixels (Figs. 10, 12, 14). The highest AGB occurred in the Yucatan Peninsula, Chiapas and Trans-Mexican Volcanic Belt. The total forest aboveground carbon (AGC) was 1966 PgC (conversion factor of 0.48), and thus higher than the NFI-calibrated AGB map (1602 PgC), the LiDAR-calibrated AGB with all LiDAR-AGB pixels (1374 PgC) as well as Mexican forest carbon stock according to FAO’s Forest Resource Assessment 2010 (1.688 PgC) [67]. The validation of the map at different scales is presented in section “Validation of forest AGB maps at different scales”.

**Fig. 13:**
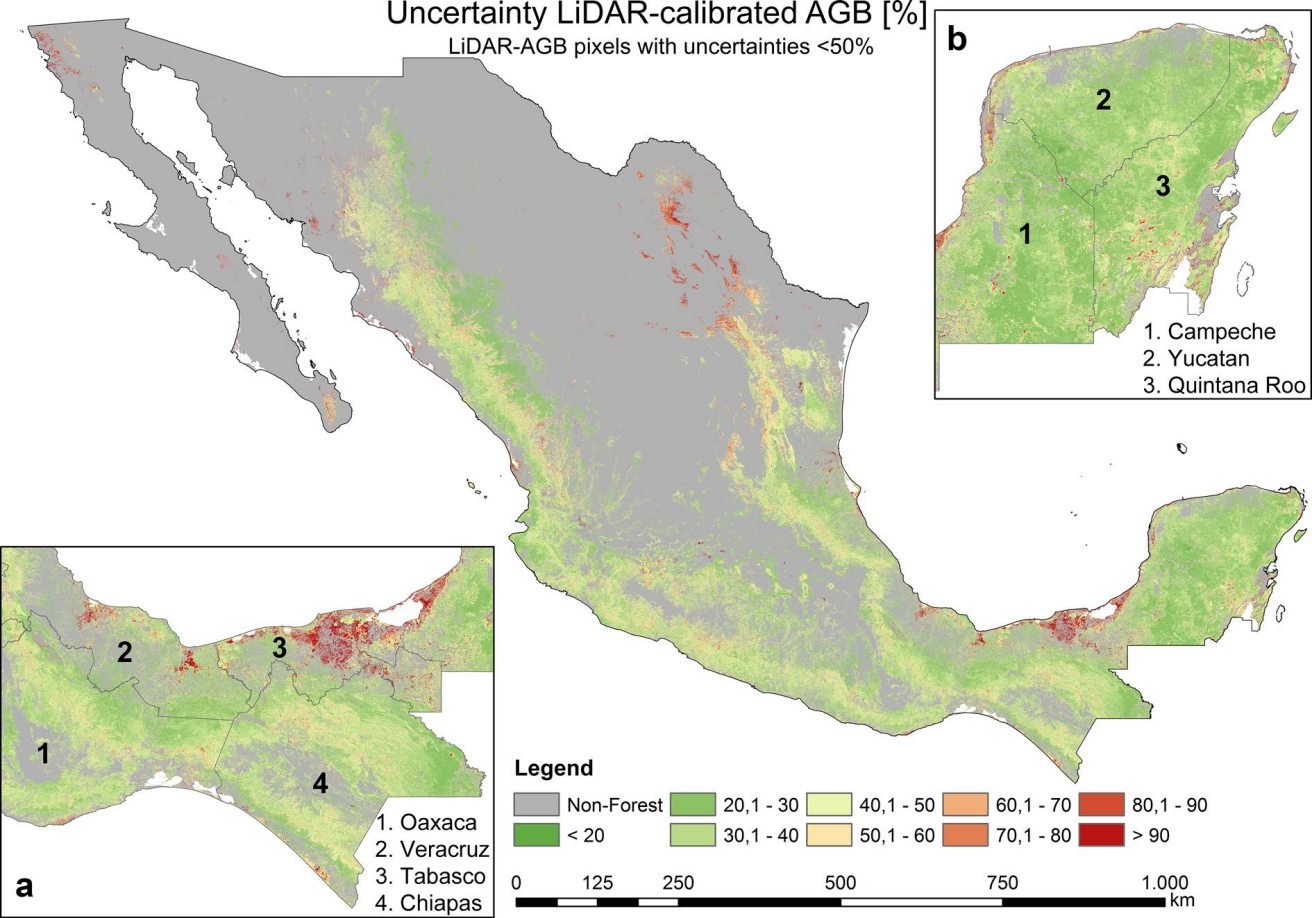
Estimated uncertainties (incl. error in field data, model prediction errors: NFI to LiDAR and LiDAR to satellite imagery) based on LiDAR-estimated AGB (with uncertainties < 50%), satellite imagery, Cubist machine learning algorithm, and Monte Carlo analyses

**Fig. 14:**
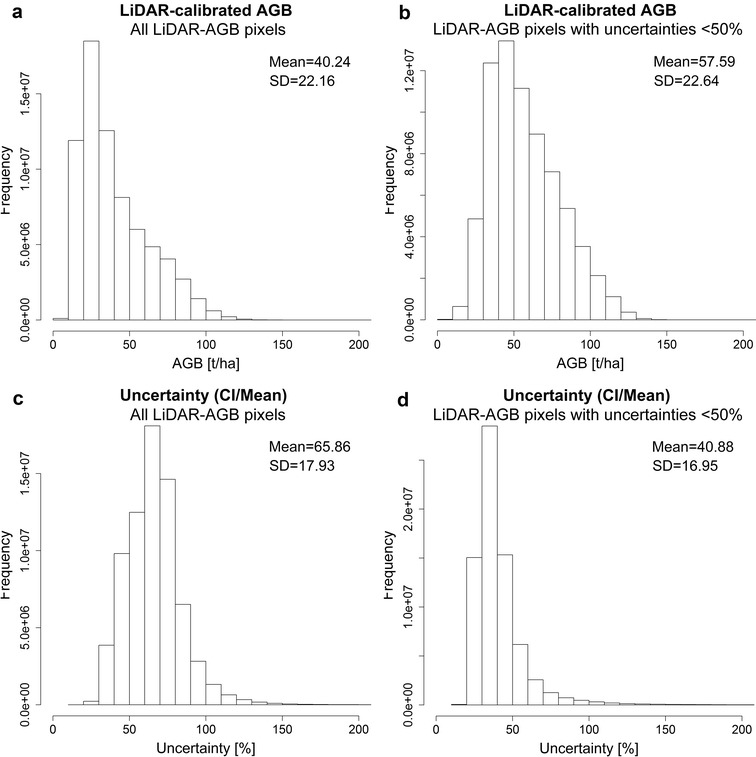
**a** Histogram of estimated national forest AGB calibrated with all LiDAR-AGB pixels; **b** histogram of estimated national forest AGB calibrated with LiDAR-AGB pixels with uncertainties < 50%; **c** histogram of uncertainties in AGB map calibrated with all LiDAR-AGB pixels; **d** histogram of uncertainties in AGB map calibrated with LiDAR-AGB pixels with uncertainties < 50%

When LiDAR-AGB_50% were used, the uncertainties in the national forest AGB map were reduced by 20–40% compared to the map calibrated with all LiDAR-AGB (Figs. 11, 13, 14c, d). In contrast to the AGB map calibrated with all LiDAR-AGB pixels, here the areas with low forest AGB showed similar uncertainties as the areas with high AGB ranging between 20 and 40%. The highest uncertainty (> 80%) in the forest AGB map calibrated with LiDAR-AGB_50% were found in the mangrove forests of Tabasco in *Pantanos de Centla*, which is similar to the NFI-calibrated AGB map (Figs. 8a, 13a). Furthermore, similar high AGB uncertainties were estimated in the north-eastern part of Mexico (state Coahuila). Possible reasons for this could be a combination of factors: (1) no LiDAR strips and only few NFI plots were available for this region, (2) and steep topography that effected radar backscatter.

As mentioned previously, the forest AGB map calibrated with all LiDAR-AGB pixels showed lower AGB values as the map calibrated with LiDAR-AGB_50%. Figure 14a and b showed that the histogram of the map calibrated with LiDAR-AGB_50% was shifted towards higher AGB values. The opposite shift towards lower uncertainties was observed in the national forest AGB map that was calibrated with LiDAR-AGB_50% (Fig. 14c, d).

### Validation of forest AGB maps at different scales

**Fig. 15:**
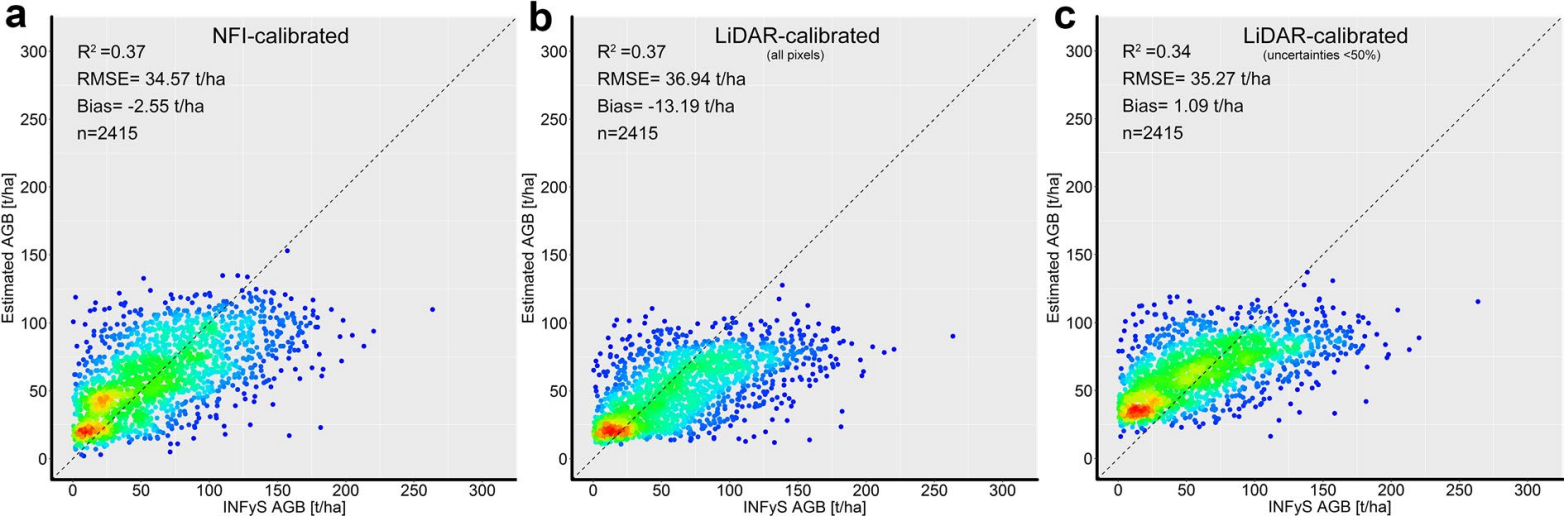
Validation at pixel scale: field-estimated AGB plotted against **a** NFI-calibrated AGB map, **b** AGB map calibrated with all LiDAR-AGB pixels, **c** AGB map calibrated with LiDAR-AGB_50%. Dotted line is the 1:1 line. Blue to red colours indicate low to high point density, respectively

The first validation was conducted at pixel level. Three maps were validated independently using forest inventory plots that were not used for model calibration (Fig. 15). The goodness-of-fit statistics were similar for all three AGB maps with similar values for R^2^ and RMSE, but a lower bias for the AGB map calibrated with LiDAR-AGB_50% (Fig. 15b, c). Obviously, all three maps underestimated the AGB in the upper range (i.e., 100–120 t/ha). This can be caused by the fact that SAR and optical imagery saturated at high AGB level, and thus became less sensitive for AGB. Furthermore, only a small amount of training data for areas with high AGB was available. This fact caused an underrepresentation of high AGB during the training process. Also, as already mentioned above, tree-based models tend to underestimate in the high range and to overestimate in the low range [34, 64]. Finally, temporal mismatch between the reference and satellite data could degrade the model performance (e.g., potential change within the field plots, as was shown in [33]).

**Fig. 16:**
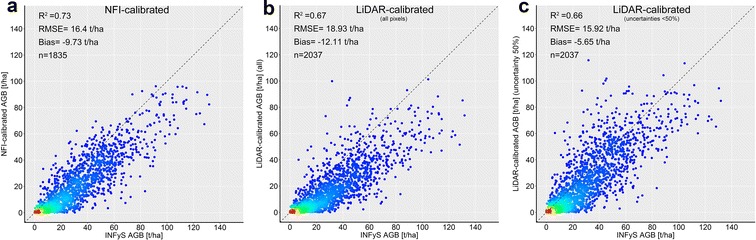
Validation at hexagon scale: field-estimated forest area weighted AGB plotted against **a** NFI-calibrated AGB map, **b** AGB map calibrated with all LiDAR-AGB pixels, **c** AGB map calibrated with LiDAR-AGB_50%. Dotted line is the 1:1 line

The second validation scale was the hexagon level. Due to spatial aggregation improved correlations were observed. All maps showed similar goodness-of-fit statistics (Fig. 16a–c). At hexagon level a slight underestimation of AGB is visible, as the most of the dots in the scatterplots were located below the 1:1 line.

**Fig. 17:**
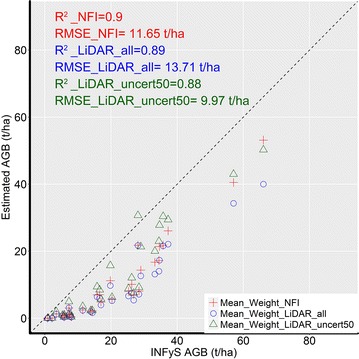
Validation at state scale: NFI- and LiDAR-calibrated AGB maps plotted against field-estimated forest area weighted AGB. Every point represents mean forest area weighted AGB for each federal state. Dotted line is the 1:1 line

At the state scale the NFI- and LiDAR-calibrated AGB maps correlated clearly with field-estimated forest area weighted AGB (Fig. 17). However, all three AGB maps showed underestimation of the forest AGB for all federal states. The smallest deviation from the 1:1 line (and the smallest RMSE) was found for the AGB map calibrated with LiDAR-AGB_50%.

## Discussion

Since NFI data are labor intensive and time consuming, and thus limited in time and space (i.e., point measurements), many remote sensing based applications use very high resolution data as reference to assess AGB. For instance, airborne LiDAR can drastically increase the number of reference data [68, 69]. In this study, we showed that LiDAR-based AGB should be used with great care for further up-scaling to satellite imagery. Although the NFI-calibrated and LiDAR-calibrated AGB maps showed similar validation results at three spatial scales, the LiDAR-calibrated AGB maps contain much larger uncertainties compared to the NFI-calibrated map. In this study, the uncertainties in the LiDAR-based AGB were much higher than the errors in the field data. These errors were propagated further to the wall-to-wall map. This resulted in very high variation of the national LiDAR-calibrated AGB. To reduce uncertainties and variations in the LiDAR-calibrated AGB map, we removed reference LiDAR-AGB pixels with high uncertainties. Consequently, the national forest AGB map calibrated with LiDAR-AGB_50% showed similar uncertainties (20–40%) as the forest AGB map calibrated with NFI data only. For further exploitation of an AGB map (e.g., decision making, modelling of C-fluxes) as well as to identify variance of the estimated AGB, a proper characterization of uncertainties and its analysis is a crucial step.

**Fig. 18:**
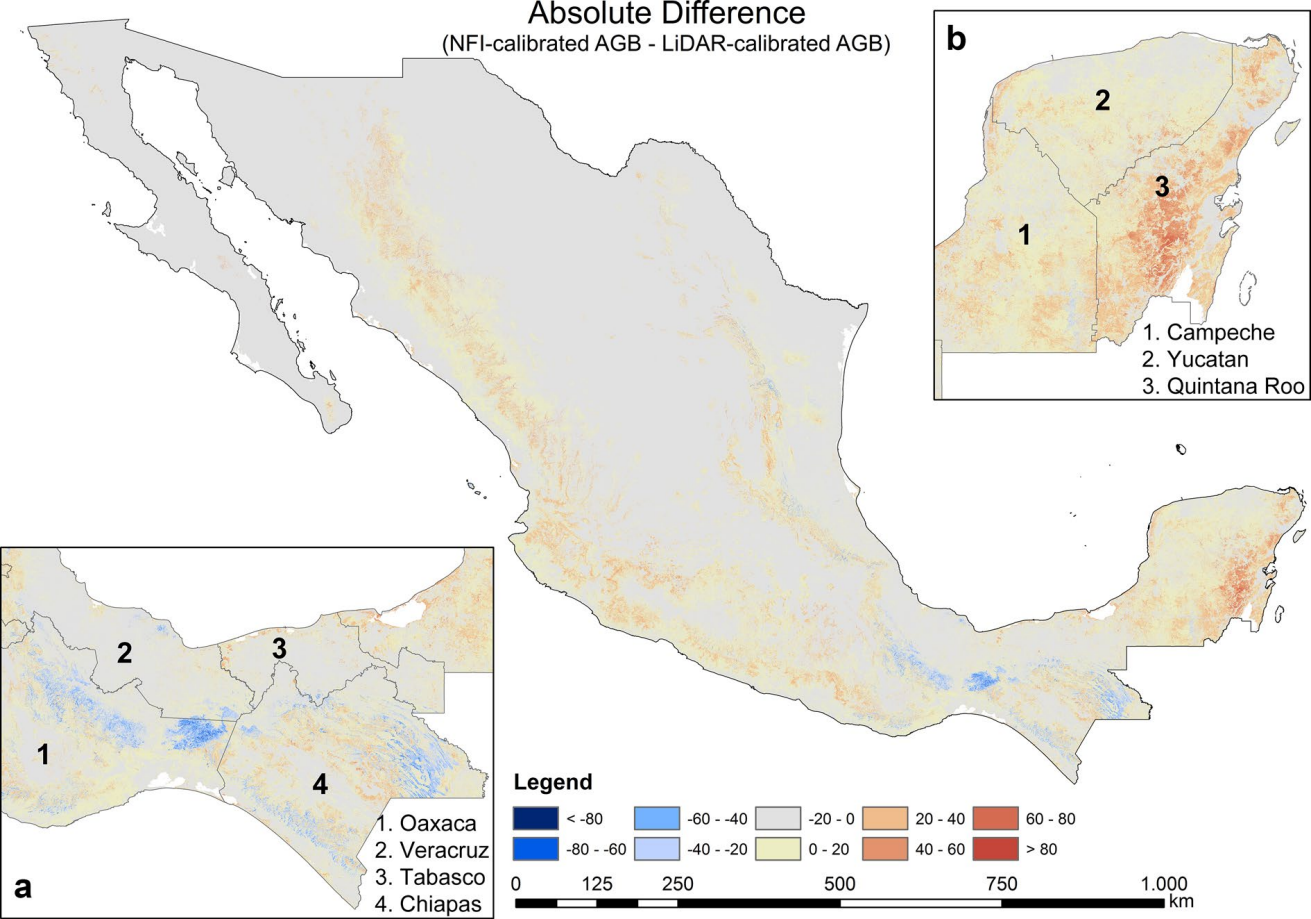
Absolute difference between the NFI- and LiDAR-calibrated AGB map (based on all LiDAR-pixels). Zoom sections **a** and **b** illustrate different spatial patterns for dense tropical forests

Furthermore, both AGB maps (NFI- and LiDAR-calibrated) showed different spatial patterns of AGB. For instance, the AGB estimates of dense tropical forests in Oaxaca and Chiapas (*Chimalapas* and *Lacandon* forests) showed a difference of 50–100 t/ha (Fig. 18a). The underestimation of AGB of the NFI-calibrated map can be caused by the fact that no or a limited number of NFI plots were available for these areas. However, we could not independently validate both AGB maps for *Chimalapas* and *Lacandon* forests due to the lack of independent reference data. Different AGB distributions were observed in the Yucatan peninsula as well (Fig. 18b), although a dense NFI network and LiDAR strips were available here.

Both AGB maps showed an underestimation at high AGB level compared with field-plot estimates (Fig. 15). The reasons as already discussed above are related to the model characteristics and the insufficient sensitivity of the satellite data for AGB at high levels (Fig. 15). These shortcomings can be partly solved through bias-correction approaches [34, 63] as well as through a greater amount of high quality reference data for high AGB intervals. Regarding satellite data the use of multi- or hyper-temporal imagery can potentially help to mitigate the signal saturation issue [70, 71]. Furthermore, the future P-band SAR mission BIOMASS [72] will provide data with a higher saturation level in forest covered areas. Eventually, considerable deviations between the AGB maps were observed in areas with steep slopes (beside the Yucatan peninsula) (Fig. 18). Accordingly, advanced terrain-correction methods for SAR imagery [e.g., 73, 74] and new accurate DEM products (e.g., TanDEM-X DEM) should be analyzed and included to further improve AGB estimations for mountainous regions.

Since there are several studies aiming at national AGB mapping for Mexico [6, 7, 12], a comprehensive comparison of the different products available is desirable. There is a clear need to support Mexico’s local authorities (e.g., CONAFOR, CONABIO) to identify and understand similarities and discrepancies of the different AGB maps as well as the source of errors.

An important issue in forest AGB mapping in Mexico is the agreement on a forest definition or a forest covered area of interest, respectively. For instance, in [6] the total AGC varied by 44% (2.21 PgC vs. 1.53 PgC), depending on whether a forest mask was applied. Rodriguez-Veiga et al. [7] applied different forest mask to calculate national forest AGC and concluded that total national forest AGC varied by 31% (lowest forest AGC of 1.47 PgC vs. highest forest AGC of 1.92 PgC). Therefore, a consistent and accurate national forest mask is crucial to assess national forest carbon stocks.

## Conclusion

The results of this study indicated that ignoring errors in the LiDAR-estimated AGB can lead to much higher uncertainties in the final wall-to-wall AGB map compared to the field to satellite up-scaling. Although the delineated forest AGB products showed similar goodness-of-fit statistics at different scales compared to the validation NFI data set (Figs. 15, 16, 17), we computed clearly higher uncertainties in the LiDAR-calibrated AGB map compared to the NFI-calibrated map. When we removed LiDAR-estimated pixel with high uncertainties, we could estimate national forest AGB with similar uncertainties as with NFI data.

Furthermore, we observed different spatial patterns of AGB in regions where no or only a limited number of NFI data were available (conservation areas in tropical forests (e.g., *Chimalapas* and *Lacandon* forests). A set of independent field plots for these regions would help to analyze and validate the presented results. Moreover, AGB at high level (> 100 t/ha) was underestimated in both modelling scenarios. We suggest that a greater number of high quality field data in dense tropical forest can mitigate this issue. Furthermore, the implementation of dense time series of satellite data will help to improve the model results. Thus, the forthcoming L-band missions (NISAR and SAOCOM) and in particular ESA’s P-band mission BIOMASS are of great interest.

Since LiDAR data can be acquired for much larger areas than field inventory data, LiDAR is an extremely important tool for repetitive reference data acquisitions over large areas, in particular in areas where the amount of NFI data are limited (e.g., restricted or inaccessible areas). Furthermore, in contrast to point measurements of field data, LiDAR captures spatial variability, which is beneficial at heterogeneous tropical forests. Nevertheless, we showed here that a two-stage up-scaling method needs to be analyzed and validated with great care. Field inventory is an essential tool to measure and observe ecological processes at local scale as it can provide a higher level of data richness when compared to LiDAR. We believe though that LiDAR can be used as an extension to NFI, for example, for areas that are difficult or not possible to access. Therefore, future research can investigate an integration of airborne LiDAR data into field inventory for forests carbon stock assessments (e.g., a trade-off between map accuracy (i.e., user requirements) and resulting costs (i.e., number of NFI and LiDAR data)).
